# The Effects of High-Dose Probiotic Supplementation on Immune Activation and Neurocognitive Disorders in People Living with HIV Undergoing Successful Antiretroviral Treatment: The Procog Study

**DOI:** 10.3390/pathogens14060568

**Published:** 2025-06-06

**Authors:** Matteo Vassallo, Margaux Zerlini, Roxane Fabre, Heloise Joly, Jacques Durant, Alain Makinson, Amandine Mauries, Jacqueline Capeau, Soraya Fellahi, Jean-Philippe Bastard, Christian Pradier, Christine Lebrun-Frenay

**Affiliations:** 1 Department of Internal Medicine/Infectious Diseases, Cannes General Hospital, 06400 Cannes, France; 2 UR2CA (URRIS), Université Côte d’Azur, 06000 Nice, France; zerlini.m@chu-nice.fr (M.Z.); joly.h@chu-nice.fr (H.J.); lebrun-frenay.c@chu-nice.fr (C.L.-F.); 3 Department of Neurology, Nice University, 06202 Nice, France; 4 Public Health Department, Archet Hospital, Nice University, 06202 Nice, France; fabre.r@chu-nice.fr (R.F.); pradier.c@chu-nice.fr (C.P.); 5 Pain Department and FHU InovPain, Nice University Hospital, Cote Azur University, 06000 Nice, France; 6 RESPECT, UR2CA, Université Côte d’Azur, 06108 Nice, France; 7 Department of Infectious Diseases, Archet Hospital, Nice University, 06202 Nice, France; durant.j@chu-nice.fr; 8 Department of Infectious Diseases, University of Montpellier, 34394 Montpellier, France; a-makinson@chu-montpellier.fr; 9 INSERM U1175, University of Montpellier, 34394 Montpellier, France; 10 Department of Geriatrics, University of Montpellier, 34394 Montpellier, France; amandine.mauries@yahoo.com; 11 Sorbonne Université-Inserm, Centre de Recherche Saint-Antoine UMR S_938, Institut Hospitalo-Universitaire de Cardio-Métabolisme et Nutrition (ICAN), 75012 Paris, France; jacqueline.capeau@inserm.fr (J.C.); soraya.fellahi@aphp.fr (S.F.); 12 Assistance Publique-Hôpitaux de Paris, Département de Biochimie-Pharmacologie, Hôpitaux Universitaires Henri Mondor, 94010 Créteil, France; jean-philippe.bastard@aphp.fr; 13 FHU-SENEC, INSERM U955 and Université Paris Est (UPEC), UMR U955, Faculté de Santé, 94000 Créteil, France

**Keywords:** HIV-associated neurocognitive disorders, probiotics, gut microbiota, immune activation

## Abstract

Background: The prevalence of HIV-associated neurocognitive disorders (HAND) remains high despite antiretroviral treatment (ART). Changes in gut microbiota and persistent immune activation have been suggested as possible causes, while the role of probiotic supplementation remains controversial. Methods: We included subjects with mild HAND and successful ART. They were randomized to receive either 6 months of high-dose probiotic supplementation or to continue with only ART. Immune activation markers and neuropsychological testing were performed at baseline and the end of follow-up. Neuropsychological testing assessed learning, episodic memory, attention/concentration, executive functions, language, information processing speed, and motor skills. Z- and T-scores were calculated for all domains but motor skills, allowing the measurement of the global deficit score (GDS). The trajectories of neuropsychological performances and immune activation markers were compared between groups. Results: From September 2020 to July 2021, 31 PWHs were included (median age 62, 73% men, CD4 744 cc/mm^3^), and 28 completed the 6-month follow-up. The characteristics of the subjects and their neuropsychological performance at baseline in the two groups were similar. At the end of follow-up, probiotics did not have any impact on immune activation markers, while they were associated with better improvement in GDS (T-score 0.0 in controls vs. −0.3 in probiotics, *p* = 0.048) and the attention/concentration test (Z-score 0.4 in controls vs. 1.2 in probiotics, *p* = 0.035). Conclusions: Oral supplementation with high-dose probiotics for 6 months did not affect systemic immune activation but was associated with improved neurocognitive performance, suggesting benefits from probiotic supplementation for mild HAND.

## 1. Introduction

Since the development and widespread use of modern antiretroviral therapy (ART), severe HIV-associated neurocognitive disorders (HAND) have considerably decreased [1,2,3,4,5].

However, altough some criticisms recently emerged about risks of HAND overestimation with the current definition, leading to suggest the more appropriate term of HIV-associated brain injury (HABI) [6], the prevalence of milder forms of HANDs, such as asymptomatic neurocognitive impairment (ANI) and mild neurocognitive disorders (MNDs), has remained stable in the ART era. These conditions have been associated with poorer quality of life, lower adherence to treatment, and increased early mortality [7,8].

Hypotheses explaining the high prevalence of neurocognitive disorders in the ART era include the direct effect of HIV on the brain, chronic immune activation, comorbidities, and gut dysbiosis [9,10]. Indeed, the gut microbiota consists of billions of microorganisms that coexist symbiotically with the human host, thereby ensuring gut barrier integrity, metabolism, and immunity [1,11]. As mucosal T-cells are among the first targets of infection, HIV has been linked to significant changes in gut microbiota. Such alterations in its composition have been associated with increased risks of microbial translocation, systemic immune activation, and neurocognitive disorders [11]. A reduction in gut microbiota diversity, an increase in pro-inflammatory species and a decrease in anti-inflammatory microorganisms are frequently observed during HIV infection [12,13]. Therefore, enriching particular species through probiotic supplementation could mitigate inflammatory responses and improve neurocognitive disorders, although data from interventional trials are still limited, and results can sometimes be contradictory [14,15,16,17].

This work aimed to measure the effect of high-dose multi-strain probiotic supplementation on immune activation and neurocognitive disorders in people living with HIV (PWHs) who have mild forms of HAND.

## 2. Materials and Methods

### 2.1. Study Design and Participants

We conducted a multicenter, randomized, prospective study involving subjects with HIV-1 infection, who were regularly monitored by one of three different Departments of Infectious Diseases in the southern region of France (Nice, Cannes, and Montpellier). The inclusion criteria were a recent diagnosis of mild HIV-associated neurocognitive disorder (HAND) and being on stable and effective ART, defined by no treatment changes and a plasma viral load of less than 50 cp/mL for at least six months before inclusion (measured with Xpert^©^ viral load or Aptima^™^ HIV Quant Dx, Hologic, Marlborough, MA, USA).

Individuals with HIV-2 infection, normal neuropsychological evaluations, dementia, recent ART modifications (<6 months), or plasma viral loads > 50 cp/mL were excluded.

This study was approved by the Limoges Ethics Committee (Comité de Protection des Personnes du Sud-Ouest et Outre-Mer IV), and patients gave written informed consent to participate.

### 2.2. Plasma Markers of Immune Activation

The following markers of immune activation were measured at inclusion and the end of follow-up: interleukin 6 (IL-6) (Roche, Cobas-8000), high-sensitivity C-reactive protein (h-CRP) (Beckman-Coulter, Immage), soluble CD14 (sCD14), soluble CD163 (sCD163), monocyte chemoattractant protein-1 (MCP-1), and soluble tumor necrosis factor receptors 1 and 2 (sTNFR-1, sTNFR-2) (Quantikine ELISA, Biotechne SA). Such markers were selected according to previous studies on inflammatory markers in PWHs [18,19,20,21].

### 2.3. Neuropsychological Testing and Definition of HAND

After completing a self-questionnaire assessing depressive symptomatology (fast-BDI) [22,23], autonomy in activities of daily living [24], and perceived state of health [25], each patient underwent a comprehensive range of neuropsychological (NP) tests (mean duration of one hour per patient), administered by a single trained neuropsychologist in Nice and Cannes, and by a second trained neuropsychologist in Montpellier. Both neuropsychologists maintained close communication to ensure a homogeneous interpretation of the NP tests.

The tests assessed a broad spectrum of cognitive domains: learning, episodic memory, attention/concentration, executive functions, language, information processing speed, and motor skills. Details of the NP tests can be found in Supplementary Materials (Table S1).

Except for motor skills, which do not have standardized scores, NP scores from each test were adjusted for age, gender, and years of education.

A global deficit score (GDS) was also calculated. Briefly, deficit scores (DSs) ranging from 0 to 5 (0 = no deficit and 5 = severe deficit) were created for each test based on the T-scores, which were derived from the Z-scores: T-score > 40 = DS of 0; T-score of 35–39 = DS of 1; T-score of 30–34 = DS of 2; T-score of 25–29 = DS of 3; T-score of 20–24 = DS of 4; T-score < 20 = DS of 5.

Then, the DS means for each domain were calculated to obtain a deficit score for each domain, excluding motor skills. These means allowed the definition of a global deficit score (GDS). According to the literature, an impaired GDS is defined by values ≥ 0.5 [26,27]

According to the American Academy of Neurology (AAN) Frascati criteria [28], PWHs with HAND were subdivided into three categories:

(1) ANI, which involves at least two cognitive domains, is documented by performance at least 1 SD below the mean on NP tests, occurring without interference in everyday functioning. The asymptomatic impairment characteristics are defined by the short version of the Instrumental Activity of Daily Living battery and by patient questioning.
(2) MND, characterized by involvement in at least two cognitive domains, is documented by performance of at least 1 SD below the mean on NP tests and is associated with mild interference in daily functioning.
(3) HIV-associated dementia (HAD) is characterized by deficits in at least two cognitive domains, evidenced by performance at least two standard deviations below the normative mean on neuropsychological tests, leading to significant interference in daily functioning.

PWHs with either ANI or MND were defined as having mild forms of impairment and continued this study, while individuals with normal performance or HAD were excluded.

After six months of follow-up, NP testing was conducted using the same criteria as those at baseline. To limit test–retest effects, parallel versions of RL.RI-16 [29,30], the Rey Complex Figure [31,32], Paced Auditory Serial Addition Test (PASAT) 3 [33], and verbal fluencies [34] were utilized.

### 2.4. Randomization

Each subject meeting the inclusion criteria was randomized into one of the following two arms:

(1) Continuing ART unchanged;
(2) Incorporating a 6-month course of high-dose oral probiotics into ART.

The selected probiotics contained 450 × 10^9^ billion bacteria per sachet, including *Lactobacillus plantarum* DSM 24730, *Streptococcus thermophilus* DSM 24731, *Bifidobacterium breve* DSM 24732, *Lactobacillus paracasei* DSM 24733, *Lactobacillus delbrueckii* subsp. *bulgaricus* DSM 24734, *Lactobacillus acidophilus* DSM 24735, *Bifidobacterium longum* DSM 24736, and *Bifidobacterium infantis* DSM 24,737 (Vivomixx^®^, Dupont, Madison, WI, USA). To minimize the risks of poor digestive tolerability, one sachet was prescribed during the first two weeks, after which the dosage was increased to two daily.

Neuropsychologists were unaware of the randomization arm used to reduce the risks of bias associated with NP testing interpretation.

### 2.5. Demographic Parameters, Background Measurements, and Dietary Habits

The following parameters were recorded for each patient and correlated with NP tests: age, gender, education, comorbid conditions (hypertension, smoking, dyslipidemia, illicit drug use, and diabetes), use of psychotropic medications (benzodiazepines, antidepressants, carbamates, and anti-epileptic drugs), CD4 T-cell count at inclusion and at the nadir, CD8 T-cell count, the CD4/CD8 ratio, time since known HIV infection, previous AIDS events, total duration of cART, time on current cART, and viral hepatitis markers.

As dietary habits can potentially impact gut microbiota, each included subject was asked about their diet (vegan, vegetarian, daily consumption of dairy products). Recent antibiotic intake was also analyzed, as it can interfere with microbiota.

### 2.6. Statistical Analysis

Frequencies and percentages were described for qualitative variables, while medians and interquartile ranges were calculated for quantitative parameters.

After describing the main characteristics of the entire population, comparisons between the probiotics and control groups were conducted using either the Chi-square or Fisher’s exact test for qualitative variables and the Wilcoxon–Mann–Whitney test for quantitative parameters.

Differences in cytokine values and NP testing at inclusion and the end of follow-up were calculated for both groups using the Wilcoxon–Mann–Whitney test. The median GDS served as the cut-off. Fisher’s test assessed its distribution according to the randomization groups. As differences were calculated from baseline to the end of follow-up, R-4.3.0 software was used to perform the statistical analysis. *p*-Values < 0.05 were considered significant.

## 3. Results

### 3.1. Characteristics of Patients at the Time of Inclusion and Trajectory of the Inflammatory Markers

From September 2020 to July 2021, 31 PWHs were included (median age 62, 73% men, CD4 at inclusion 744 cc/mm^3^; see Table 1), while 10 patients were excluded because NP testing was normal in 9 cases, and 1 subject had HAD. According to the AAN classification for HAND, 93% of subjects had ANI and 7% MND.

The characteristics of patients at inclusion were similar regarding probiotic randomization, except for higher cow’s milk consumption observed in the probiotics group (Table 1). Current ART included two nucleoside transcriptase inhibitors (NRTIs) + one integrase strand transfer inhibitor (INSTI) for 10 subjects, one non-nucleoside transcriptase inhibitor (NNRTI) + one INSTI for 8 individuals, two NRTIs + one NNRTI for 5 subjects, and one NRTI + one INSTI for 4 subjects, while four PWHs had another type of ART. Furthermore, no differences were found in hepatitis B or C co-infection, previous illicit drug use, diabetes, hypertension, alcohol intake, or smoking.

Three subjects (10%) did not complete the 6-month follow-up period due to poor tolerance to probiotics (mainly diarrhea) or a lack of motivation to continue this study.

Out of the 31 PWHs, 28 (90%) completed the follow-up period (mean duration 6.4 +/− 1.8 months). Neither antibiotic intake nor changes in dietary habits were recorded during the follow-up. Their inflammatory markers at inclusion were similar in both groups, and their trajectory did not differ at the end of the follow-up, except for hCRP, which increased in the probiotics group but remained in the normal range (Table 2 and Table 3).

### 3.2. Trends in Neuropsychological Performance

At baseline, NP testing results did not differ between the two groups. In particular, in the probiotics and control groups, the GDS values at inclusion were 0.6 and 0.4 in the probiotics and control groups, respectively.

After six months of follow-up, no differences were found between the two groups regarding language, executive functions, learning, memory, or information processing speed. However, a trend indicated better attention and working memory performance in the probiotics group (Table 4). Furthermore, patients receiving probiotics showed significantly improved GDS compared to the control group (Table 4).

**Figure 1:**
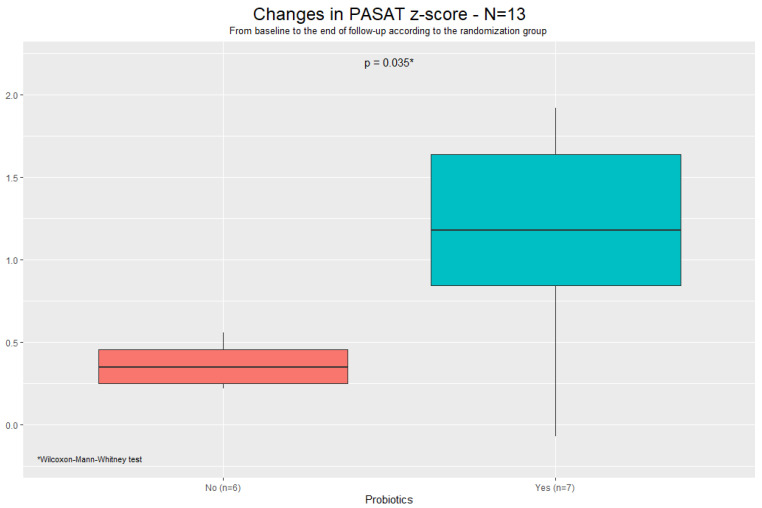
PASAT: Paced Auditory Serial Addition Test 3.

Moreover, although only 13 subjects completed the PASAT3 at both NP assessments, those on probiotics showed a significant improvement in their performance (Z-score 0.4 in controls vs. 1.2 in probiotics, *p* = 0.035, Figure 1).

The two groups presented no differences in motor skills (Table 4).

## 4. Discussion

We found that oral supplementation with high-dose probiotics for 6 months did not affect systemic immune activation in PWHs with mild neurocognitive disorders. However, probiotic intake was associated with improved neurocognitive performance, measured by the global deficit score (GDS). Moreover, we also observed an improvement in the PASAT score within the probiotics group, which is generally associated with attention skills.

These results align with those of Ceccarelli et al., who used the same oral supplementation and demonstrated improvements in cognitive function and CNS immune activation [14].

Several hypotheses can be formulated to explain the lack of effect of probiotics on immune activation markers observed in our study. Firstly, the gut microbiome plays a complex immunomodulatory role in several immune cells, such as monocytes, lymphocytes, dendritic cells, and epithelial intestinal cells, therefore including both innate and adaptative immunity, which can occur both locally and systemically [35]. In this work, we measured several markers of systemic immune activation in plasma but did not assess gut-derived metabolites, permeability, neuro-inflammatory markers, or mucosal immunity. Indeed, microbial metabolites, such as short-chain fatty acids, serotonin, tryptophan metabolites, and lipopolysaccharides, have previously been associated with an unhealthy gut microbiota [36,37,38]. Restoring a healthy microbiota through probiotic supplementation may be associated with either a decrease in such metabolites or with the improvement of mucosal immunity, thereby reducing immune activation independently of the measured cytokines. Moreover, although our work included a few patients with symptomatic HAND, we did not analyze markers in the cerebrospinal fluid (CSF) as was performed in the Ceccarelli study, which showed a benefit of probiotics on monocyte activation [15]. In any case, we did not measure the impact of probiotics on lymphocyte and monocyte markers of activation, which have been associated with HIV neuropathogenesis in several studies [39,40,41]. Additionally, the lack of effect on inflammatory markers may be related to the small sample size, which was partially constrained by the COVID-19 pandemic during the study period.

Although we did not find any significant effects of probiotics on individual cognitive domains, the interventional arm was associated with improved sustained attention, as measured by PASAT, and neurocognitive performance, assessed using GDS. These results are promising, considering that effective interventions for subjects with HAND and successful ART remain scarce. Indeed, while ART has dramatically improved the severity of neurocognitive disorders in untreated PWH [42], randomized trials involving treated PWHs with HAND, which modified ART to enhance its penetration in the brain, have failed to demonstrate any benefits [43,44]. Furthermore, data regarding the impact of early cognitive training on brain function are limited, although they are encouraging [45,46].

If confirmed by more extensive prospective studies, our work suggests that improving gut microbiota diversity may have a potential benefit on neurocognitive disorders. Indeed, the intestinal microbiome regulates the maturation of the mucosal immune system, including lymph nodes, lamina propria, and epithelial cells, thereby ensuring intestinal homeostasis and inhibiting inflammation [11]. In cases of dysbiosis, this balance is disrupted, resulting in profound alterations in mucosal immunity and an increased risk of inflammation [11]. Furthermore, altering the gut mucosal barrier allows many bacteria-derived products to enter the bloodstream, altering blood–brain barrier permeability and precipitating local inflammation. Indeed, Ceccarelli et al. demonstrated that probiotic supplementation was associated with reduced CNS immune activation, as measured by neopterin levels [14].

This study’s limitations include its small sample size and the fact that the randomization was not blinded. However, as mentioned earlier, neuropsychologists were unaware of the inclusion arm when NP testing was performed. Furthermore, analysis of gut microbiota before and after probiotic supplementation would have provided additional insights into their composition and effects on neurocognitive performance. Lastly, based on only six domains, the GDS may not fully capture the entire NP performance. Nevertheless, motor skills, which were excluded from the GDS, did not differ between groups.

## 5. Conclusions

In conclusion, although there were no effects on immune activation markers in this pilot study, we found that high-dose multi-strain probiotic supplementation may be beneficial for neurocognitive performance in PWHs with HAND. More extensive prospective studies are needed to clarify their effects on the brain and inflammation.

## Figures and Tables

**Table 1:** Characteristics of subjects included.

		Total—N = 31		Randomization				
				No Probiotics—*n* = 15		Probiotics—*n* = 16		
	*n*	Median	[Q1; Q3]	Median	[Q1; Q3]	Median	[Q1; Q3]	*p*-Value *
Age at inclusion (years)	31	62.0	[57.0; 71.0]	63.0	[56.0; 73.0]	60.5	[57.0; 66.3]	0.553
Years of study (*n*)	30	9.0	[9.0; 12.0]	9.0	[8.5; 10.5]	11.0	[9.0; 12.0]	0.191
CD4 count at inclusion (cc/mm^3^)	31	744.0	[558.5; 816.0]	612.0	[506.0; 803.5]	773.0	[662.3; 880.5]	0.188
CD8 count at inclusion (cc/mm^3^)	31	840.0	[541.5; 1105.5]	733.0	[486.5; 1105.5]	891.5	[604.3; 1072.3]	0.892
Nadir CD4 (cc/mm^3^)	30	246.0	[133.3; 396.3]	184.0	[47.5; 372.0]	252.0	[189.0; 460.0]	0.161
Viral load peak (cp/mL)	21	82,640.0	[8100.0; 150,000.0]	91,000.0	[12,200.0; 100,500.0]	53,835.0	[11,250.0; 197,250.0]	0.973
Years on ART (*n*)	23	19.0	[16.0; 26.0]	18.5	[17.0; 24.8]	24.0	[15.0; 26.0]	1.000
ART regimens received (*n*)	22	7.0	[4.3; 12.0]	8.0	[4.5; 10.8]	6.5	[4.5; 12.8]	0.921
Months on last ART (*n*)	30	25.0	[13.8; 38.8]	25.0	[16.5; 49.5]	24.0	[11.0; 31.0]	0.443
Daily consumption of yogurt (*n*)	24	1.0	[1.0; 2.0]	1.0	[1.0; 2.0]	2.0	[1.0; 2.0]	0.657
Daily consumption of cheese (*n*)	24	1.0	[1.0; 2.0]	1.0	[1.0; 2.0]	1.0	[1.0; 2.0]	0.898
Daily consumption of milk (centiliters)	25	0.0	[0.0; 100.0]	0.0	[0.0; 0.0]	60.0	[0.0; 162.5]	0.027
	***n***	***n***	**(%)**	***n***	**(%)**	***n***	**(%)**	***p*** -Value **
Sex	31							1.000
Female		8	(25.8)	4	(50.0)	4	(50.0)	
Men		23	(74.2)	11	(47.8)	12	(52.2)	
AIDS	31							0.220
No		24	(77.4)	10	(41.7)	14	(58.3)	
Yes		7	(22.6)	5	(71.4)	2	(28.6)	
Vegetarian diet	31							-
No		28	(100.0)	14	(50.0)	14	(50.0)	
Missing		*3*		1		2		
Vegetalian diet	31							-
No		29	(100.0)	14	(48.3)	15	(51.7)	
Missing		2		1		1		

* Wilcoxon–Mann–Whitney test. ** Either Khi or Fisher tests.

**Table 2:** Inflammatory markers at inclusion (*n* = 28).

	Total—N = 28		Probiotics				
			No—*n* = 14		Yes—*n* = 14		
	Median	[Q1; Q3]	Median	[Q1; Q3]	Median	[Q1; Q3]	*p*-Value *
Il6 (pg/mL)	2.0	[1.5; 2.8]	2.6	[1.5; 4.0]	1.7	[1.5; 2.3]	0.188
hCRP (mg/L)	1.2	[0.5; 2.0]	1.6	[0.9; 2.4]	0.7	[0.4; 1.4]	0.135
sCD14 (ng/mL)	2473.8	[2104.3; 3473.4]	2473.8	[2137.3; 3104.1]	2657.7	[2056.4; 3518.9]	0.910
sCD163 (pg/mL)	386.7	[300.5; 491.4]	376.8	[307.7; 415.3]	386.7	[295.1; 505.9]	0.839
MCP-1 (pg/mL)	329.9	[273.7; 382.7]	340.9	[275.0; 381.0]	326.8	[281.8; 371.5]	0.667
sTNFR1 (pg/mL)	1182.8	[1003.7; 1671.8]	1182.8	[1054.0; 1736.3]	1249.5	[819.4; 1524.4]	0.482
sTNFR2 (pg/mL)	2267.6	[1809.8; 3068.0]	2271.5	[1892.6; 2642.7]	2267.6	[1581.2; 3200.4]	0.701

* Wilcoxon–Mann–Whitney test; IL6: interleukin 6. sCD14: soluble CD14. sCD163: soluble CD163. MCP-1: monocyte chemoattractant protein-1. sTNFR1: soluble tumor necrosis factor receptor 1. sTNFR2: soluble tumor necrosis factor receptor 2.

**Table 3:** Trends for inflammatory markers from baseline to the end of follow-up according to randomization.

	Probiotics				
	No—*n* = 14		Yes—*n* = 14		
	Median	[Q1; Q3]	Median	[Q1; Q3]	*p*-Value *
Differences M6-D0					
Il-6 (pg/mL)	−0.1	[−1.1; 0.0]	0.0	[−0.2; 1.1]	0.114
hCRP (mg/L)	−0.4	[−0.8; −0.0]	0.3	[−0.3; 2.3]	0.031
sCD14 (ng/mL)	−216.4	[−748.1; 259.1]	−38.9	[−751.9; 365.5]	0.804
sCD163 (pg/mL)	−1.2	[−23.1; 112.4]	54.8	[31.8; 131.4]	0.194
MCP-1 (pg/mL)	−5.0	[−51.0; 29.4]	−3.6	[−62.0; 26.1]	1.000
sTNFR1 (pg/mL)	−70.6	[−351.7; −25.4]	133.3	[−115.6; 431.4]	0.094
sTNFR2 (pg/mL)	−88.1	[−393.8; 236.0]	190.7	[−156.9; 630.1]	0.125

* Wilcoxon–Mann–Whitney test. IL6: interleukin 6. sCD14: soluble CD14. sCD163: soluble CD163. MCP-1: monocyte chemoattractant protein-1. STNFR1: soluble tumor necrosis factor receptor 1. STNFR2: soluble tumor necrosis factor receptor 2.

**Table 4:** Effects of probiotics on NP performance.

		Probiotics				
		No—*n* = 14		Yes—*n* = 14		
	*n*	Median	[Q1; Q3]	Median	[Q1; Q3]	*p*-Value *
Trajectory from baseline to month 6						
Language DS	28	0.0	[−0.8; 0.0]	−0.5	[−0.5; 0.0]	0.230
Attention/working memory DS	20	0.0	[−0.2; 0.3]	−0.2	[−0.4; 0.0]	0.079
Executive function DS	28	−0.1	[−0.3; 0.4]	0.0	[−0.3; 0.0]	0.944
Learning DS	28	0.0	[−0.1; 0.4]	−0.1	[−0.4; 0.3]	0.366
Memory DS	20	−0.1	[−0.4; 0.0]	−0.3	[−0.6; 0.2]	0.711
Information processing speed DS	28	0.0	[−0.8; 1.0]	0.0	[−1.0; 0.0]	0.199
GDS	28	0.0	[−0.2; 0.1]	−0.3	[−0.4; −0.1]	0.048
IADL score	28	0.0	[−0.8; 0.8]	0.0	[0.0; 0.0]	0.936
BDI-FS score	28	−0.5	[−3.8; 0.8]	−0.5	[−2.8; 0.0]	0.871
SF-36						
Physical component summary	28	−0.3	[−6.9; 6.1]	−3.8	[−9.8; 2.2]	0.748
Mental component summary	28	1.2	[−3.2; 12.1]	1.05	[−12.5; 6.0]	0.667
	***n***	***n***	**(%)**	***n***	**(%)**	*p*-Value **
GDS	28					0.023
≤−0.137 (median)		4	(28.6)	10	(71.4)	
>−0.137 (median)		10	(71.4)	4	(28.6)	
Finger tapping M6	28					0.445
<4		5	(35.7)	7	(50.0)	
4		9	(64.3)	7	(50.0)	
Luria M6	28					0.705
<4		8	(57.1)	7	(50.0)	
4		6	(42.9)	7	(50.0)	

* Wilcoxon–Mann–Whitney test. ** Fisher’s exact test. DS: domain score. GDS: global deficit score. IADL: Instrumental Activities of Daily Living. BDI-FS: Beck Depression Inventory-Fast Screen. SF-36: Short Form-36.

## Data Availability

The original contributions presented in this study are included in this article/Supplementary Materials; further inquiries can be made to the corresponding author.

## Supplementary Materials

The following supporting information can be downloaded at https://www.mdpi.com/article/10.3390/pathogens14060568/s1, Table S1: Description of neuropsychological tests used. References cited in Supplementary Materials [47,48,49].
