# Supplementary material for: The Effects of High-Dose Probiotic Supplementation on Immune Activation and Neurocognitive Disorders in People Living with HIV Undergoing Successful Antiretroviral Treatment: The Procog Study
Source: Pathogens. 2025 Jun 6;14(6):568. doi: 10.3390/pathogens14060568 (PMC12196004; doi:10.3390/pathogens14060568)
Supplement: Supplementary file 1 [file pathogens-14-00568-s001.zip › pathogens-3645271-supplementary.pdf]

Supplementary Materials

Table S1. Description of neuropsychological tests used.

**Learning:**	the immediate recall scores and the scores from trials 1 to 3 (free and cued recall) are controlled for encoding, according to the RL/RI-16 [29], which assesses the different processes of verbal episodic long-term memory (encoding, learning, storage, retrieval, consolidation). Then, the patient is asked to recall as many words as possible from a list of sixteen. This includes three short-term free and cued recall trials, one delayed free and cued recall, and a recognition task.
**Memory:**	delayed recall scores on the RL/RI-16 (free and cued); the richness of recall score for the Rey Complex Figure [31], a complex drawing copied by the patient five minutes earlier, assess visual long-term memory; and the forward digit span task (Wechsler, 2008), which consists of recalling a series of digits immediately after their oral presentation at a rate of one per second, assess verbal short-term memory.
**Information processing speed:**	through the Computerized Speed Cognitive Test (CSCT) [47], a rapid computerized test where the participant must enunciate as many numbers as possible corresponding to symbols shown on a computer within 90 seconds, based on a correspondence visible throughout the test and randomly generated to avoid learning effects.
**Attention and working memory:**	are measured by the time and error scores of Trail Making Test TMT-A, a part of the TMT [48], which involves connecting circles containing digits in ascending order; the time score of TMT-B, the second part of the test, where the instruction is to alternately connect circles containing digits and letters in ascending and alphabetical order; the score from the reversed digit span test [49], which measures verbal working memory by having the participant recall series of numbers presented orally at a rate of one per second, in reverse order; and the score from the PASAT test in its 3-second version [33], an auditory test assessing sustained attention and working memory where the subject hears 61 numbers at a rate of one every three seconds and must, after each number presented, add it to the previous number heard and provide the total.
**Executive functions:**	using the type of copy of the Rey figure [31], which indicates planning ability; the number of perseverative errors in TMT-B [48]; and the difference between the time and number of errors in TMT-B and TMT-A, measuring mental flexibility ability.
**Language:**	through formal verbal fluency [33], in which the subject was required to generate as many different words as possible starting with the letter P (R in parallel version) in two minutes, excluding proper nouns and words from the same lexical family, and categorical fluency, where the

	participant needed to name as many animals (fruits in parallel version) names as possible within two minutes.
**Motor skills:**	through the Luria motor sequences, where the patient is asked to perform as many consecutive slice-fist-palm motor sequences as possible using the non-dominant hand within 10 seconds, along with the finger tapping test, in which the index finger taps against the middle finger of the non-dominant hand as many times as possible within 5 seconds.
